# CMR techniques and findings in children with myocarditis: a multicenter retrospective study

**DOI:** 10.1186/1532-429X-16-S1-P119

**Published:** 2014-01-16

**Authors:** Puja Banka, Santosh Uppu, Matthew A Harris, Keren Hasbani, Wyman W Lai, Marc Richmond, Sohrab Fratz, Supriya Jain, Tiffanie Johnson, Shiraz A Maskatia, Jimmy C Lu, Margaret M Samyn, David J Patton, Andrew J Powell

**Affiliations:** 1 Boston Children's Hospital, Boston, Massachusetts, USA; 2 Mount Sinai Hospital, New York, New York, USA; 3 Children's Hospital of Philadelphia, Philadelphia, Pennsylvania, USA; 4 Advocate Children's Hospital, Park Ridge, Illinois, USA; 5 Morgan Stanley Children's Hospital of New York-Presbyterian, New York, New York, USA; 6 Deutsches Herzzentrum München, Munich, Germany; 7 Maria Fareri Children's Hospital at Westchester Medical Center, Valhalla, New York, USA; 8 Riley Hospital For Children, Indianopolis, Indiana, USA; 9 Texas Children's Hospital, Houston, Texas, USA; 10 C.S. Mott Children's Hospital, Ann Arbor, Michigan, USA; 11 Medical College of Wisconsin, Milwaukee, Wisconsin, USA; 12 Alberta Children's Hospital, Calgary, Alberta, Canada

## Background

CMR is increasingly used to diagnose myocarditis in adults but its use in pediatric-age pts is not well established. We sought to describe the clinical presentation, CMR imaging protocols, CMR findings, and outcomes in a multicenter cohort of children with myocarditis.

## Methods

A retrospective review was conducted among 12 institutions from 3 countries. All pts meeting the following criteria were included: 1) age < 21 years, 2) ultimate clinical diagnosis of myocarditis by the referring physicians, 3) CMR examination within 30 days of presentation, and 4) no congenital heart disease. Clinical data and test results, including CMR findings, were abstracted from the medical record.

## Results

**Figure 1:**
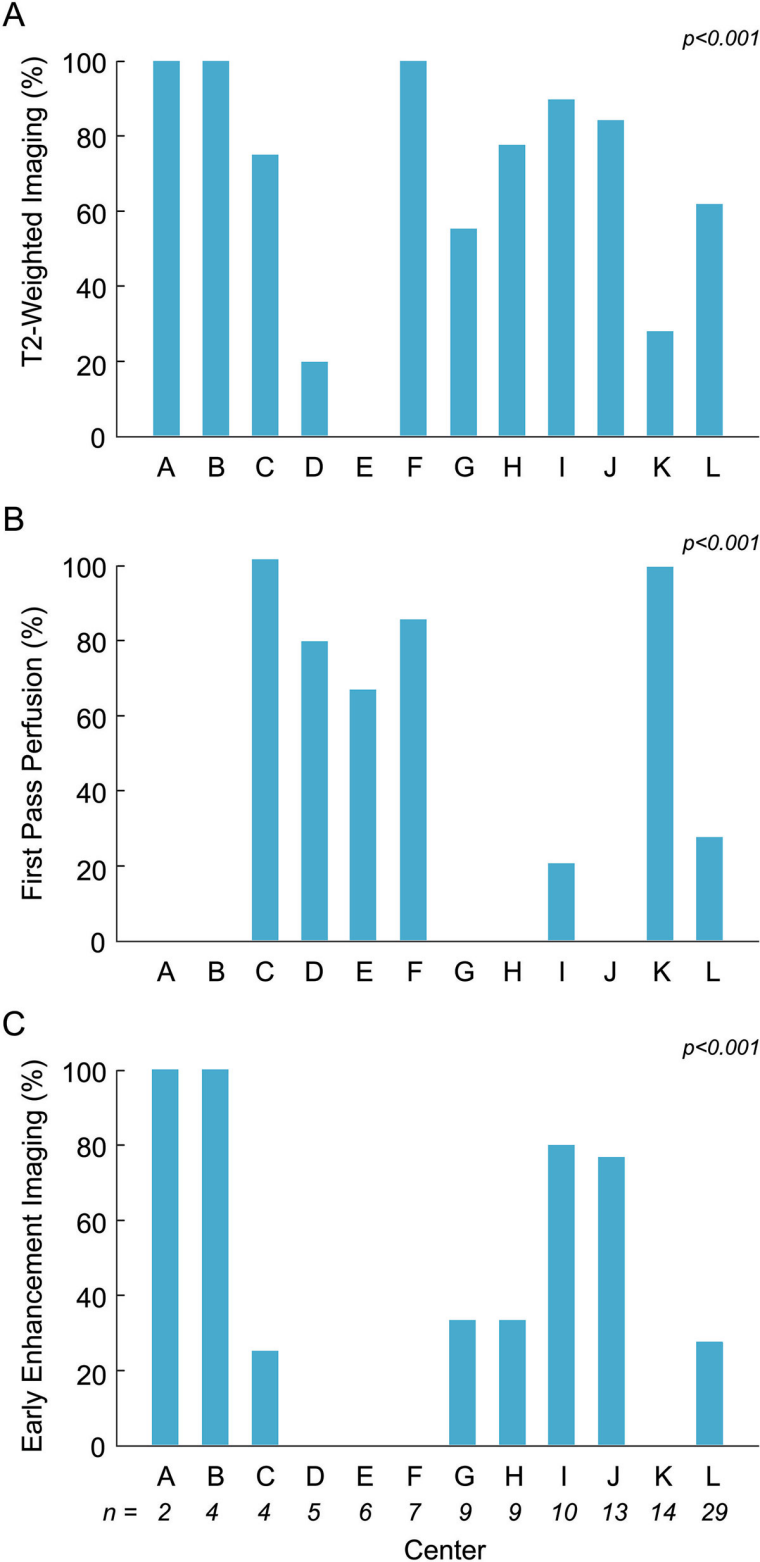
**Percent of CMR studies at each participating center (A-L) in which T2W (A), FPP (B), and EGE imaging (C) was performed**. Number of pts enrolled from each institution is given at the bottom of panel (C).

A total of 112 pts (median age 16 yrs (0-20)) met inclusion criteria. On echo at presentation, 22 pts (20%) had moderate or severe left ventricular (LV) dysfunction, and 20 (24%) had regional wall motion abnormalities. Endomyocardial biopsy was performed in 24 pts (21%), of which 12 were positive based on histology and 5 borderline. Percent of pts undergoing biopsy was similar across institutions (p = 0.32). Median time from presentation to CMR was 2 days (0-30), and 107 (96%) were inpatients. Sedation was used in 15 CMR studies (14%), and inotropic support in 16 (14%). Median LV ejection fraction (EF) was 56% (10-74) with 22% having EF < 45%. Median right ventricular EF was 55% (16-72) with 9% having EF < 40%. T2-weighted imaging (T2W) was performed in 71 studies (66%) and was abnormal in 49 (69%). First pass contrast perfusion (FPP) imaging was performed in 42 studies (45%) and was abnormal in 4 (10%). T1-weighted imaging for early gadolinium enhancement (EGE) was performed in 35 studies (37%) and was abnormal in 19 (51%). Late gadolinium enhancement (LGE) imaging was performed in all studies, and was abnormal in 93 (83%) with the following reported distributions: 89% subepicardial or midwall, 6% patchy, 3% sub-endocardial, 1% transmural, and 1% diffuse. The CMR study was interpreted as positive for myocarditis in 96 pts (87%), negative in 11 (10%), and equivocal in 4 (4%), yielding a sensitivity of 86% for an ultimate clinical diagnosis of myocarditis. There was significant practice variation in the use of T2W, FPP, and EGE imaging among the participating institutions (Figure 1). At a median follow-up time from CMR of 6 mo (0.2-217), all patients were alive and 3 had undergone cardiac transplantation, all of whom had CMR studies positive for myocarditis.

## Conclusions

This is the largest study to date describing the CMR findings in children with myocarditis. The CMR techniques used, from most to least common, were LGE, T2W, FPP, and EGE. Abnormalities were most often seen with LGE followed by T2W, EGE, and FPP. There was significant practice variation in the CMR protocol between institutions. The information from this study should be useful in planning a prospective study to evaluate the diagnostic and predictive performance of CMR in children with suspected myocarditis.

## Funding

None.

